# Post-Operative Cognitive Impairment: A Cognitive Epidemiology Perspective

**DOI:** 10.3390/jintelligence10010018

**Published:** 2022-03-11

**Authors:** Insa Feinkohl

**Affiliations:** Max-Delbrück-Center for Molecular Medicine in the Helmholtz Association (MDC), Molecular Epidemiology Research Group, D-13125 Berlin, Germany; insa.feinkohl@mdc-berlin.de

**Keywords:** cognitive epidemiology, cognitive ageing, pre-morbid IQ, education, post-operative cognitive impairment, surgery

## Abstract

Cognitive epidemiology investigates cognitive predictors of health and disease outcomes. Post-operative cognitive impairment is a common complication of surgery but has been neglected as a health outcome in cognitive epidemiology research. This is despite the fact that knowledge of cognitive predictors of post-operative cognitive impairment can be utilized for risk stratification, informed decision-making (in elective surgery), and personalized care of patients during the postoperative period. In this narrative review, the current literature on cognitive predictors of post-operative cognitive impairment and gaps therein are summarized.

## 1. Cognitive Epidemiology

Cognitive epidemiology is a growing research field that addresses individual differences in intelligence at its peak level in early adulthood, i.e., prior to any ageing- or disease-associated declines, (also termed ‘pre-morbid IQ’) as a candidate risk factor for health and disease outcomes (Deary and Batty 2007; Deary 2012). Luckily to epidemiologists, short of actual measurement (like in birth cohorts), pre-morbid IQ can be estimated ‘post-hoc’ at any point across the lifespan by performance on vocabulary tests (Bright and van der Linde 2020). This is because vocabulary is a ‘crystallized’ ability (Cattell 1963) and as such immune to declines (Crawford et al. 2001). Self-report proxies of pre-morbid IQ such as socioeconomic status (SES), household income, education, and occupation (e.g., Cheng and Furnham 2019), which together with pre-morbid IQ can be referred to as ‘cognitive reserve’ parameters (Stern et al. 2020), are also frequently used. Numerous studies have demonstrated links of a lower achievement on these measures with an increased risk of later negative health outcomes including premature mortality (Batty et al. 2007; Calvin et al. 2011; Melchior et al. 2006), cancer (Batty et al. 2009), accelerated cognitive ageing (Caamaño-Isorna et al. 2006; Huang et al. 2020b), and COVID-19 infection (Batty et al. 2020).

## 2. Cognitive Epidemiology in a Surgical Setting

Surgery, particularly in older age, comes with a risk of complications, and some of them are of a cognitive nature. Post-operative delirium (POD) is characterized by agitation or apathy and affects around 19–28% of patients during the days after surgery (Ho et al. 2021); post-operative cognitive dysfunction (POCD) describes a decline in cognitive test performance across surgery that can last weeks to years. At 3 to 6 months, POCD can be detected in around 10–25% of patients (Borchers et al. 2021). This article aims to provide a narrative review of the currently limited evidence on the cognitive epidemiology of post-operative cognitive impairment. To this end, associations of cognitive reserve parameters with POD and POCD risk from studies of any design will be summarized, differences that emerge for these two outcomes will be drawn out, and knowledge gaps will be highlighted for future systematic examination in view to stimulate further research.

Of note, any epidemiological research in a surgical setting is highly complicated as compared with population-based settings. In the latter, one can take advantage of large cohorts that track participants over time and monitor them for occurrence of disease outcomes. Participants are recruited after detailed pre-planning, at a time when they are (presumably) of good general health and are happy to donate their time for research upon recruitment and (often) several times during the years or decades thereafter. In surgical settings, in contrast, patients are approached at a time of psychological distress and poor health and are followed up for a limited time period only, usually up to hospital discharge or a few months after surgery. Drop-out is common and sample sizes are often small. A focus is mainly on hard facts, such as blood-based biomarkers or medication plans, rather than more or less easily quantifiable (and in terms of immediate survival certainly less relevant) parameters such as pre-morbid IQ or education. It is, therefore, difficult to apply cognitive epidemiology research methods developed in population-based settings to the analysis of post-operative complications, such as POD or POCD. The studies reviewed here reflect this difficulty. They also vary considerably in terms of study design, follow-up period, surgery/anesthesia type, and patient characteristics, many of which can be risk factors in their own right, but will not be at the focus of this work. In addition, studies differ in their definitions of POCD, which can be based on various statistical techniques applied to scores from repeat neuropsychological testing or brief screening instruments. For POD, in contrast, comparability between studies is aided by the use of standardized instruments such as Confusion Assessment Method (CAM) or an ICU-specific version (CAM-ICU). A majority of studies reviewed here also had not set out to target the cognitive epidemiology of POD or POCD but reported patients’ cognitive reserve parameters (most frequently education) in ‘baseline characteristics’ tables comparing ‘POD’ with ‘no POD’ or ‘POCD’ with ‘no POCD’ groups. This lack of statistical adjustment is not necessarily a problem in this context; however: other than genetic or early-life exposures, no confounding factor could be expected to lead to both a low pre-morbid IQ and POCD for instance, giving rise to a spurious statistical association in absence of a true link between the two. In this respect, cognitive epidemiology takes on a special kind of role in the investigation of health and disease: cognitive reserve exposures can be expected to stand at (or near) the outset of a causal chain leading up to a disease outcome in older age.

### 2.1. The Cognitive Epidemiology of POD

#### 2.1.1. Education and POD

The evidence on education and POD risk appears mixed. In the Successful Aging after Elective Surgery (SAGES) study of 566 over 70-year-old patients undergoing major non-cardiac surgery in the US, POD defined by a combination of CAM and chart review occurred in 24% of patients. Self-reported education was not associated with POD risk (Cavallari et al. 2016, 2017; Cizginer et al. 2017; Hshiesh et al. 2017; Inouye et al. 2016; Tripp et al. 2021; Vasunilashorn et al. 2017). Another large study, the PAWEL study based in Germany (Deeken et al. 2022), as well as smaller cohort studies from Europe, Asia, and South America support these null results (Jiang et al. 2017; Kazmierski et al. 2013; Mahanna-Gabrielli et al. 2020; Puustinen et al. 2016; Ristescu et al. 2021; Sauer et al. 2017; Seo et al. 2014; Xue et al. 2016), whereas other investigations do suggest a low education as a risk factor for POD (Dworkin et al. 2016; Gu et al. 2021; Guan et al. 2021; Huang et al. 2021; Ma et al. 2021; McAlpine et al. 2008; Ordóñez-Velasco and Hernández-Leiva 2021; Schoen et al. 2011; Sprung et al. 2017; Su et al. 2019; Tenpaku et al. 2021; Tobar et al. 2018). Interestingly, one recent Chinese study of 323 laryngectomy patients reported evidence in the opposite direction. Here, 85.7% of patients who developed POD (defined according to CAM) had a high school degree, whereas only 61.4% of patients without POD had a high school degree; the remaining patients had attained less than high school. The difference was statistically significant and suggested a protective association of a low education with a reduced POD risk (Wang et al. 2019).

Systematic reviews mirror this incoherent evidence. One that focused on cardiac surgery settings concluded that a low education was not a risk factor for POD (Gosselt et al. 2015); another describes a low education as a risk factor for POD (Bilotta et al. 2013) but cites two studies of which one finds no such association (DeCrane et al. 2011) and the other did not assess education (Gusmao-Flores et al. 2012). A number of other systematic reviews on risk factors for POD neglected education altogether (Aitken et al. 2017; Oh et al. 2015; Raats et al. 2016), which could be an oversight or may result from education infrequently being associated with POD. Clearly, the evidence on education and POD risk warrants systematic review and meta-analysis for quantification of any potential associations.

#### 2.1.2. Occupation and POD

In the SAGES study, the only study reporting on occupation and POD, complexity and managerial demands in patients’ self-reported longest held occupation were not associated with POD risk, both across the total study sample and when analyzed for females and males separately (Saczynski et al. 2014).

#### 2.1.3. Estimated Pre-Morbid IQ and POD

Pre-morbid IQ apparently has been assessed in only two studies in the context of POD. In a study of 37 older cardiac surgery patients in the UK, a trend short of statistical significance was observed for a lower National Adult Reading Test (NART) in patients who developed POD compared with those who did not (Brown et al. 2011). In SAGES, patients scoring higher on an estimate of pre-morbid IQ (Wechsler Test of Adult Reading, WTAR) were at a reduced POD risk in univariate (Cizginer et al. 2017) and multivariate-adjusted analysis (Saczynski et al. 2014), and with a substantial effect size: each 0.5 standard deviation higher WTAR score was associated with a 38% reduced POD risk when controlling for age, sex, ethnicity, and English as a second language. The association appeared to be driven by females in particular (Saczynski et al. 2014). An analysis of a subsample of the first 300 patients enrolled into SAGES had already indicated a trend in this direction (Fong et al. 2015).

#### 2.1.4. Composites and POD

One way to operationalize exposures for cognitive epidemiology research is to calculate composite (e.g., latent) variables of reserve, which can avoid parameter-specific measurement error (Deary and Johnson 2010). In SAGES, a latent composite was calculated from estimated pre-morbid IQ, education, and self-reported lifetime engagement in cognitive activities. The resulting composite (thus far) appears to have been assessed as a moderator of the relationship of post-operative C-reactive protein concentrations with POD risk only (Cizginer et al. 2017) rather than as a potential predictor of POD in its own right.

### 2.2. The Cognitive Epidemiology of POCD

#### 2.2.1. Education and POCD

Compared with POD, the evidence on education and POCD is more well-described and consistent. In a 2017 meta-analysis of 15 studies from Europe, USA, Australia, and Asia, with follow-up periods of one day to six months after surgery (Feinkohl et al. 2017), education was strongly inversely associated with POCD risk: each one additional year of education was associated with a 10% reduced POCD risk. Meta-regression indicated that the finding was independent of follow-up period, sample age, % males, and surgery type, suggesting that the association of education with reduced POCD risk may be universal. Similar observations were made for education as a categorical variable: patients with more than high school education were overall at 29% reduced POCD risk compared with patients who only attained a high school degree (Feinkohl et al. 2017). To name one landmark study included in the meta-analysis, the International Study of Post-Operative Cognitive Dysfunction (ISPOCD 1), found that among 1011 older surgical patients recruited in eight European countries and the USA, patients with more than a high school education had even a halved risk of POCD at one week after surgery compared with patients with less than high school education (Moller et al. 1998). Studies from various European and Asian countries and the USA that were not included in the 2017 meta-analysis (Grichnik et al. 1999) or published thereafter (Bendikaite and Vimantaite 2020; Ehsani et al. 2020; Huang et al. 2020a; Klinger et al. 2019; Li et al. 2020; Wang et al. 2018; Yuhe et al. 2020; Zhang et al. 2019; Zhou et al. 2020) too, consistently indicate that patients with a lower educational level are at increased POCD risk at discharge (Zhang et al. 2019) within days of surgery (Ehsani et al. 2020; Li et al. 2020; Zhao et al. 2020; Zhou et al. 2020) but also during six to 12 weeks after surgery (Grichnik et al. 1999; Yuhe et al. 2020). The fact that observations extend to several months after surgery is important because studies on POCD with longer follow-up periods offer preferred scope for conclusions: during shorter follow-up periods immediate effects of surgery on brain function including a potential influence of POD can prevail. Null results on education and POCD appear relatively rare and coincide with a low statistical power (Claes et al. 2018; Li et al. 2019; Ni et al. 2015; Relander et al. 2020).

#### 2.2.2. Occupation and POCD

A single study has compared occupational background between patients with and without POCD. In a cohort of middle-aged to older CABG patients in Finland, no association of occupation (categorized as ‘manual routine’, ‘qualified manual’, ‘nonmanual’) and POCD risk was observed (Relander et al. 2020). However, the study of 99 patients (13% with POCD) was again limited by a low statistical power.

#### 2.2.3. Estimated Pre-Morbid IQ and POCD

Only two studies, both set in Australia, appear to have investigated estimated pre-morbid IQ as a stand-alone exposure. Both found no association with POCD risk. One of older atrial fibrillation ablation patients used the NART and found no association with POCD at two days or at three months after surgery (Medi et al. 2013). Another study of 53 knee surgery patients aged ≥50 years used a revision of the WTAR (Test of Premorbid Functioning, Pearson 2003) as an estimate of pre-morbid IQ and found no association with POCD at six months (Scott et al. 2017).

#### 2.2.4. Composites and POCD

In a unique study of 42 middle-aged to older CABG patients, Ropacki et al. (Ropacki et al. 2007) calculated a composite from estimated pre-morbid IQ, education, occupation, as well as ethnicity, sex, and region of country in the US. The reasoning behind inclusion of the latter three parameters was not well substantiated and may have led to the observation that the composite was not associated with risk of POCD at hospital discharge. In fact, a statistically non-significant trend of a lower POCD risk in the ‘low cognitive reserve’ group (55.5%) compared with the ‘high cognitive reserve’ group (77.3%) was observed in this study (Ropacki et al. 2007).

### 2.3. Summary

A low education can be considered a firm risk factor for POCD. Although we can only speculate as to underlying mechanisms, lifestyle-associated parameters such as lifetime exposure to smoking and metabolic dysfunction may be plausible mediators enhancing the vulnerability of the brain to surgery effects. For POD, the evidence for associations with education appears more mixed and warrants systematic assessment including meta-analysis and meta-regression to determine whether or not any between-study differences in design, instruments, or patient characteristics may account for differences in results. As it currently stands, it appears that the link of education with POD may be weaker as compared with its association with POCD, or it may be absent. This pattern adds to mounting evidence indicating that POD and POCD, despite both having symptoms related to brain dysfunction, are in fact distinct conditions (Daiello et al. 2019) with a distinct epidemiology. The evidence on occupation and pre-morbid IQ in POD/POCD risk is extremely limited at present and further studies are needed to evaluate their roles.

## 3. Conclusions and Outlook

Based on the evidence presented here, cognitive reserve parameters may warrant consideration in any epidemiological studies on lifespan risk factors for post-operative cognitive impairment. They could act as potential confounders: patients with low education may be at increased risk of developing chronic diseases for instance (Wraw et al. 2015) as well as POCD (Feinkohl et al. 2017), which could lead to a spurious statistical association between chronic disease and POCD. To aid epidemiological research into POD and POCD, we thus clearly need to strengthen our research efforts to gain a fuller understanding of their cognitive epidemiology.

Although cognitive screening instruments such as the Mini Mental State Examination (MMSE) are not ideal for definition of cognitive impairment, in busy clinics with limited time resources, they are certainly preferable to having no cognitive assessment at all (Deary 2021) and could be a way to boost POCD research in particular. Of note, pre-morbid IQ estimated by vocabulary remains stable across surgery (Brown et al. 2011). Thus, it is conceivable that future studies could measure this (and other) cognitive reserve markers *after* surgery, at a time when detailed assessments may be felt to be less burdensome to patients, and still produce valid results.

Ultimately, once we have a better understanding of the cognitive epidemiology of post-operative cognitive impairment, we can apply this knowledge for risk stratification and personalized medicine. We will be able to empower patients for informed decision-making in elective surgery settings and can additionally monitor at-risk individuals for cognitive deficits during the post-operative period. With cognitive reserve (to some extent) modifiable, pre-operative intervention programs could be set up and tested. We, thus, need to recruit large cohorts of surgical patients and test not only for POD and POCD, but additionally (before or after surgery) collect data on a diverse set of cognitive reserve markers. Some rare studies in this direction, such as SAGES, have already shown us how.

## Data Availability

Not applicable.
